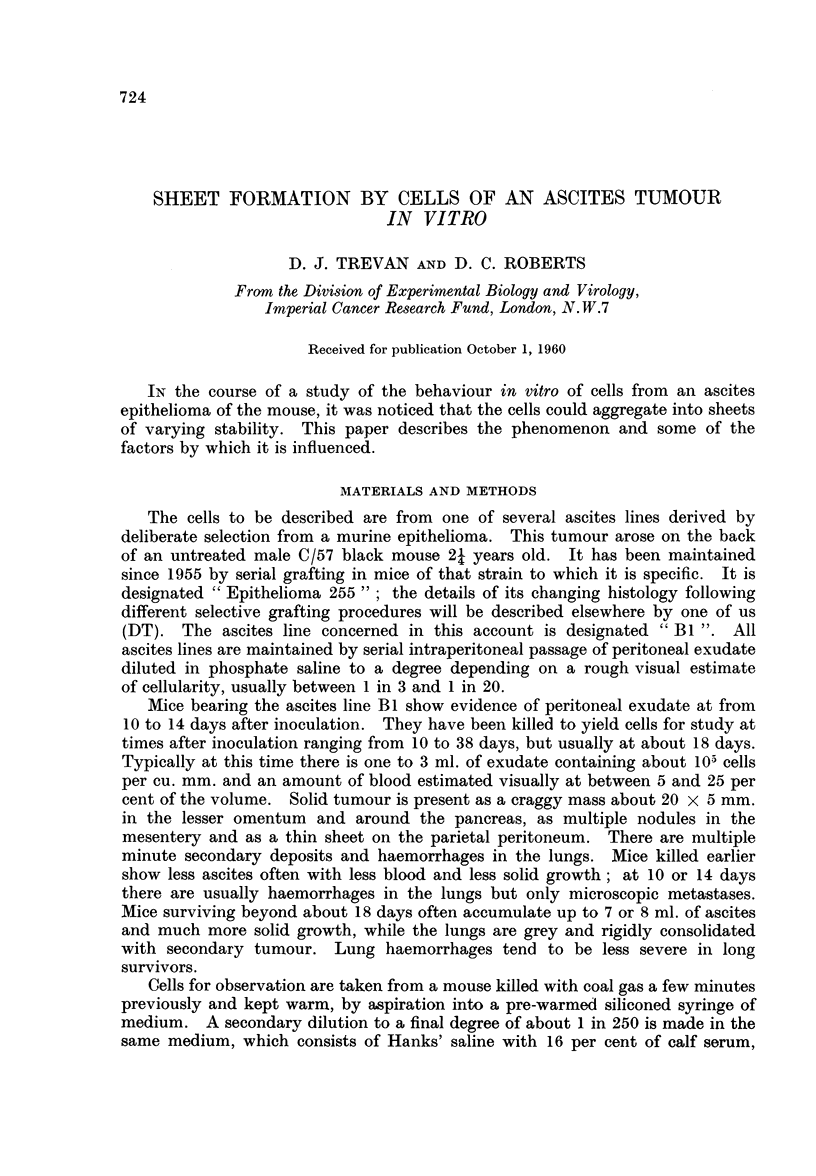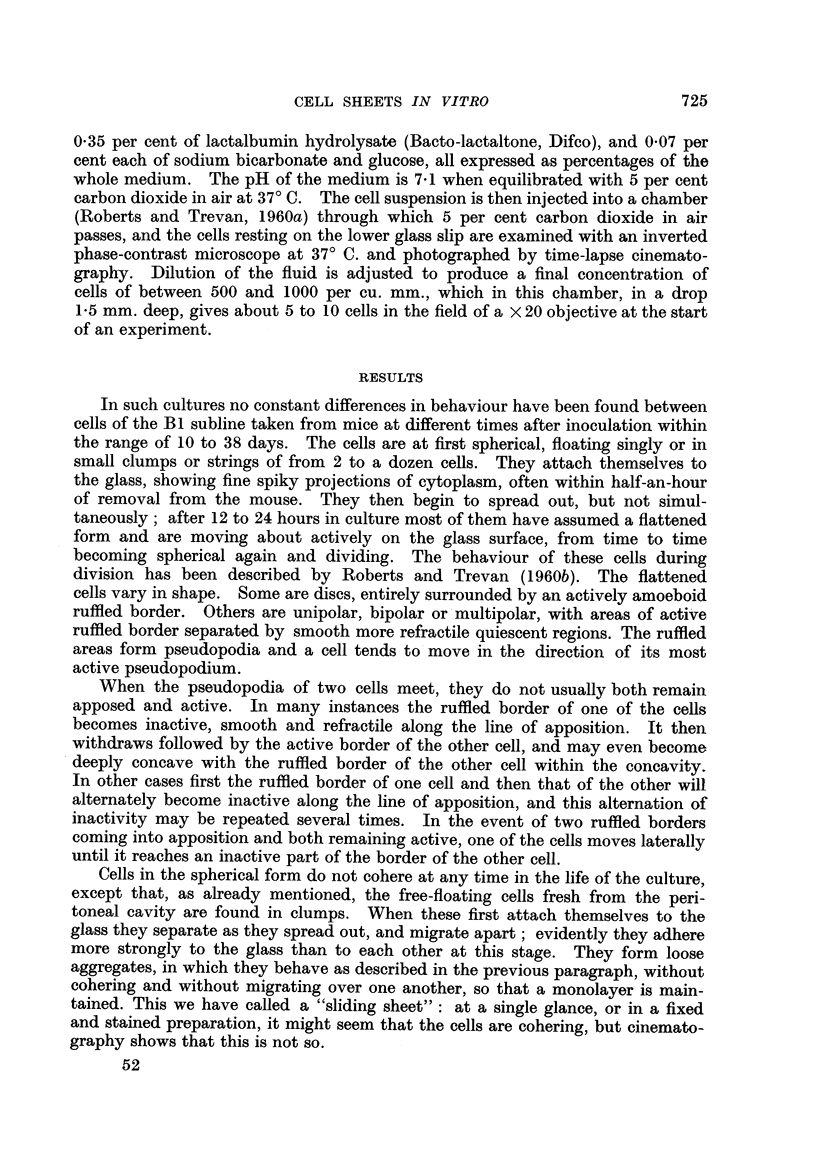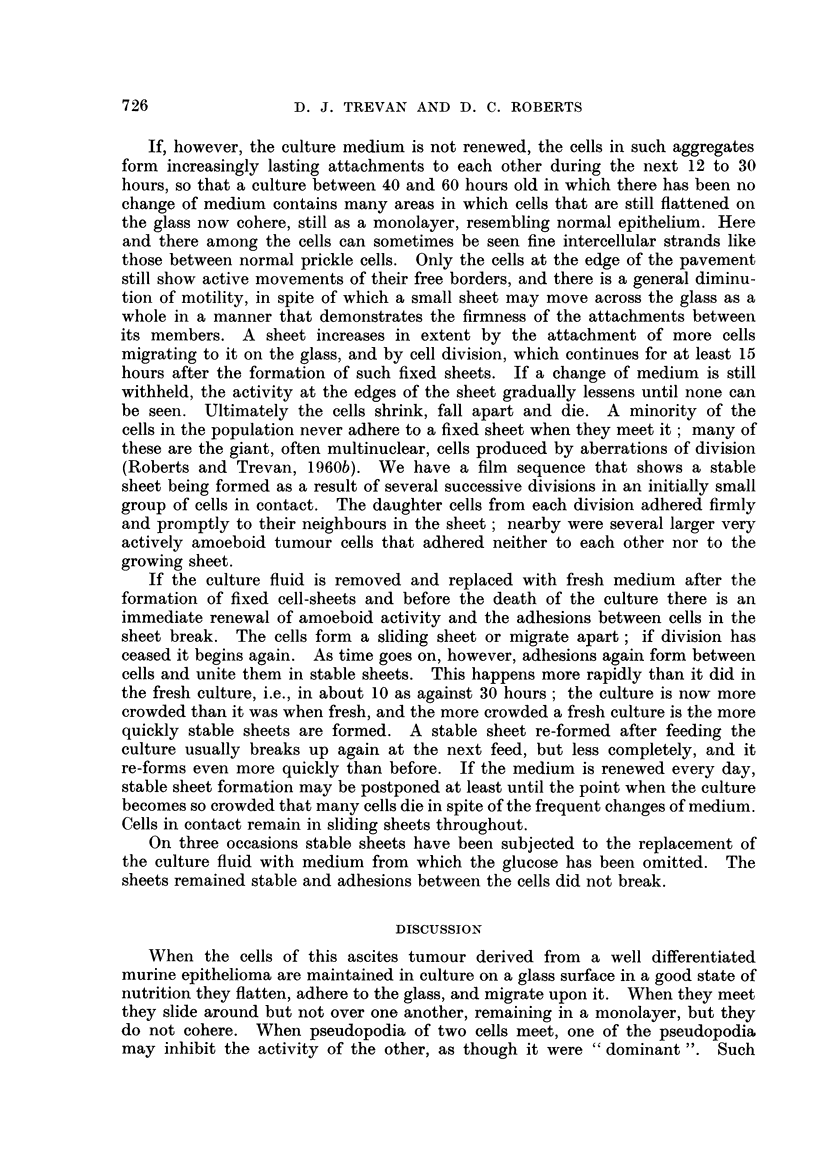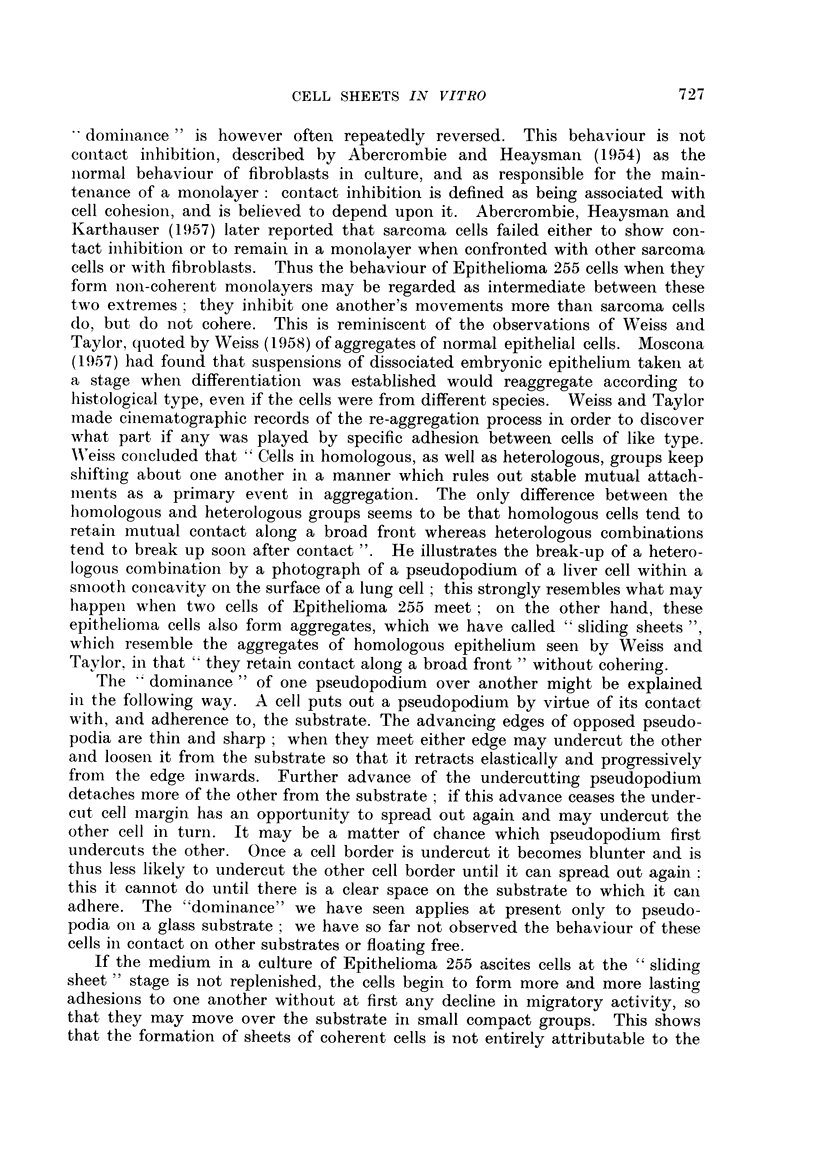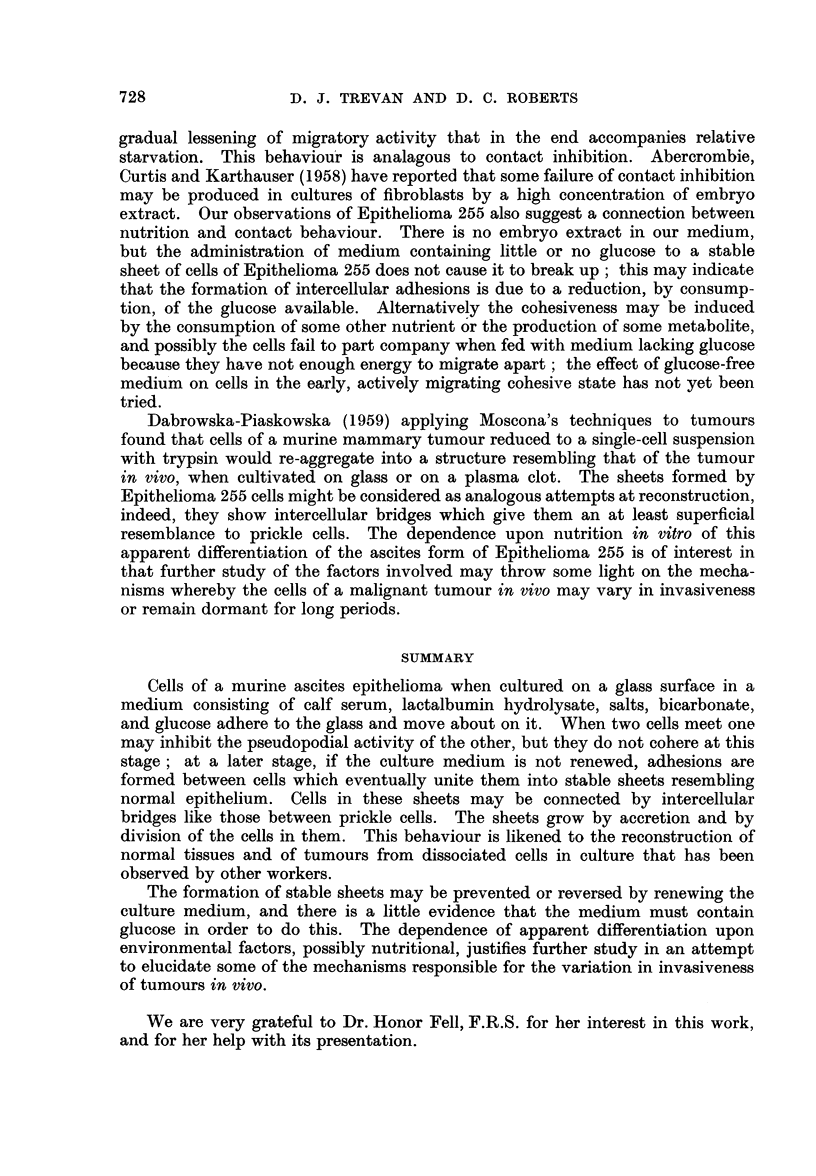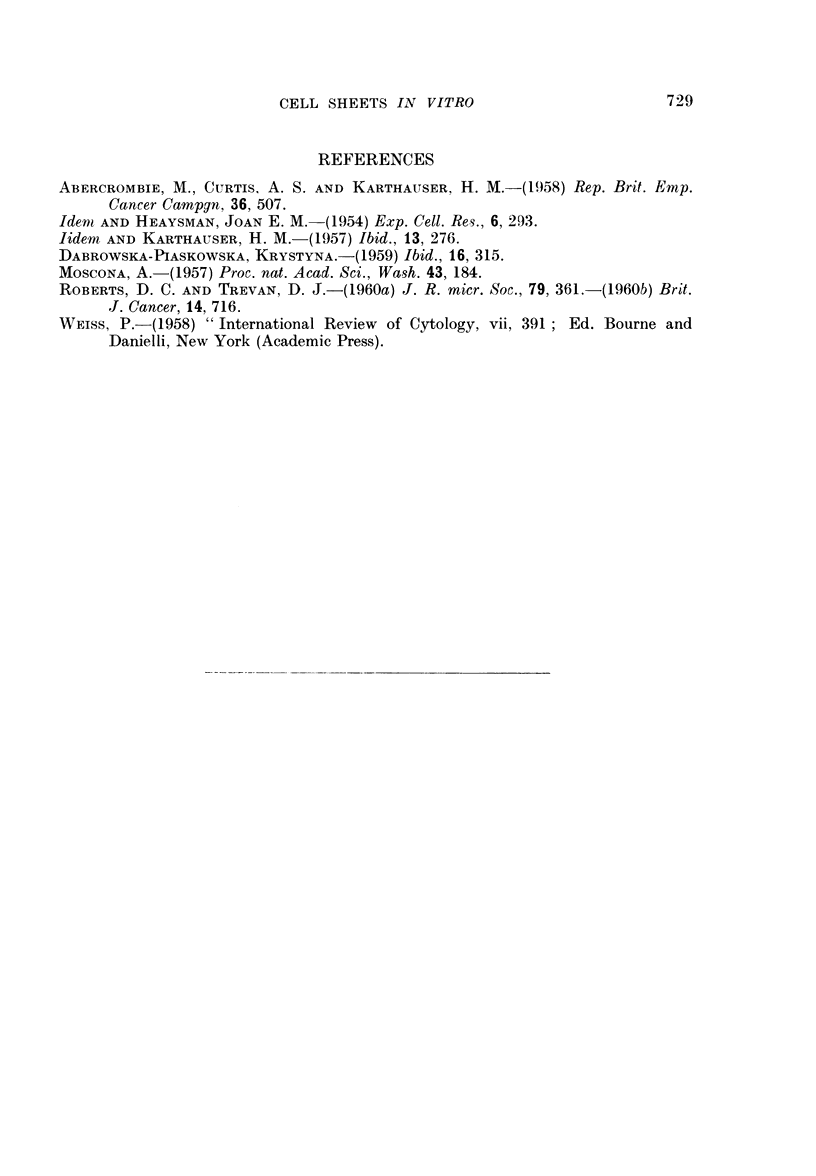# Sheet Formation by Cells of an Ascites Tumour in vitro

**DOI:** 10.1038/bjc.1960.82

**Published:** 1960-12

**Authors:** D. J. Trevan, D. C. Roberts


### SHEET FORMATION BY CELLS OF AN ASCITES TUMOUR *IN VITRO*

D. J. TREVAN AND D. C. ROBERTS

*From the Division of Experimental Biology and Virology, Imperial Cancer Research Fund, London, N.W.7*

Received for publication October 1, 1960

IN the course of a study of the behaviour *in vitro* of cells from an ascites epithelioma of the mouse, it was noticed that the cells could aggregate into sheets of varying stability. This paper describes the phenomenon and some of the factors by which it is influenced.

### MATERIALS AND METHODS

The cells to be described are from one of several ascites lines derived by deliberate selection from a murine epithelioma. This tumour arose on the back of an untreated male C/57 black mouse $2\frac{1}{4}$ years old. It has been maintained since 1955 by serial grafting in mice of that strain to which it is specific. It is designated " Epithelioma 255 " ; the details of its changing histology following different selective grafting procedures will be described elsewhere by one of us (DT). The ascites line concerned in this account is designated " B1 ". All ascites lines are maintained by serial intraperitoneal passage of peritoneal exudate diluted in phosphate saline to a degree depending on a rough visual estimate of cellularity, usually between 1 in 3 and 1 in 20.

Mice bearing the ascites line B1 show evidence of peritoneal exudate at from 10 to 14 days after inoculation. They have been killed to yield cells for study at times after inoculation ranging from 10 to 38 days, but usually at about 18 days. Typically at this time there is one to 3 ml. of exudate containing about $10^5$ cells per cu. mm. and an amount of blood estimated visually at between 5 and 25 per cent of the volume. Solid tumour is present as a craggy mass about 20 $\times$ 5 mm. in the lesser omentum and around the pancreas, as multiple nodules in the mesentery and as a thin sheet on the parietal peritoneum. There are multiple minute secondary deposits and haemorrhages in the lungs. Mice killed earlier show less ascites often with less blood and less solid growth ; at 10 or 14 days there are usually haemorrhages in the lungs but only microscopic metastases. Mice surviving beyond about 18 days often accumulate up to 7 or 8 ml. of ascites and much more solid growth, while the lungs are grey and rigidly consolidated with secondary tumour. Lung haemorrhages tend to be less severe in long survivors.

Cells for observation are taken from a mouse killed with coal gas a few minutes previously and kept warm, by aspiration into a pre-warmed siliconed syringe of medium. A secondary dilution to a final degree of about 1 in 250 is made in the same medium, which consists of Hanks' saline with 16 per cent of calf serum,

0·35 per cent of lactalbumin hydrolysate (Bacto-lactaltone, Difco), and 0·07 per cent each of sodium bicarbonate and glucose, all expressed as percentages of the whole medium. The pH of the medium is 7·1 when equilibrated with 5 per cent carbon dioxide in air at 37° C. The cell suspension is then injected into a chamber (Roberts and Trevan, 1960*a*) through which 5 per cent carbon dioxide in air passes, and the cells resting on the lower glass slip are examined with an inverted phase-contrast microscope at 37° C. and photographed by time-lapse cinematography. Dilution of the fluid is adjusted to produce a final concentration of cells of between 500 and 1000 per cu. mm., which in this chamber, in a drop 1·5 mm. deep, gives about 5 to 10 cells in the field of a $\times 20$ objective at the start of an experiment.

### RESULTS

In such cultures no constant differences in behaviour have been found between cells of the B1 subline taken from mice at different times after inoculation within the range of 10 to 38 days. The cells are at first spherical, floating singly or in small clumps or strings of from 2 to a dozen cells. They attach themselves to the glass, showing fine spiky projections of cytoplasm, often within half-an-hour of removal from the mouse. They then begin to spread out, but not simultaneously ; after 12 to 24 hours in culture most of them have assumed a flattened form and are moving about actively on the glass surface, from time to time becoming spherical again and dividing. The behaviour of these cells during division has been described by Roberts and Trevan (1960*b*). The flattened cells vary in shape. Some are discs, entirely surrounded by an actively amoeboid ruffled border. Others are unipolar, bipolar or multipolar, with areas of active ruffled border separated by smooth more refractile quiescent regions. The ruffled areas form pseudopodia and a cell tends to move in the direction of its most active pseudopodium.

When the pseudopodia of two cells meet, they do not usually both remain apposed and active. In many instances the ruffled border of one of the cells becomes inactive, smooth and refractile along the line of apposition. It then withdraws followed by the active border of the other cell, and may even become deeply concave with the ruffled border of the other cell within the concavity. In other cases first the ruffled border of one cell and then that of the other will alternately become inactive along the line of apposition, and this alternation of inactivity may be repeated several times. In the event of two ruffled borders coming into apposition and both remaining active, one of the cells moves laterally until it reaches an inactive part of the border of the other cell.

Cells in the spherical form do not cohere at any time in the life of the culture, except that, as already mentioned, the free-floating cells fresh from the peritoneal cavity are found in clumps. When these first attach themselves to the glass they separate as they spread out, and migrate apart ; evidently they adhere more strongly to the glass than to each other at this stage. They form loose aggregates, in which they behave as described in the previous paragraph, without cohering and without migrating over one another, so that a monolayer is maintained. This we have called a "sliding sheet" : at a single glance, or in a fixed and stained preparation, it might seem that the cells are cohering, but cinematography shows that this is not so.

If, however, the culture medium is not renewed, the cells in such aggregates form increasingly lasting attachments to each other during the next 12 to 30 hours, so that a culture between 40 and 60 hours old in which there has been no change of medium contains many areas in which cells that are still flattened on the glass now cohere, still as a monolayer, resembling normal epithelium. Here and there among the cells can sometimes be seen fine intercellular strands like those between normal prickle cells. Only the cells at the edge of the pavement still show active movements of their free borders, and there is a general diminution of motility, in spite of which a small sheet may move across the glass as a whole in a manner that demonstrates the firmness of the attachments between its members. A sheet increases in extent by the attachment of more cells migrating to it on the glass, and by cell division, which continues for at least 15 hours after the formation of such fixed sheets. If a change of medium is still withheld, the activity at the edges of the sheet gradually lessens until none can be seen. Ultimately the cells shrink, fall apart and die. A minority of the cells in the population never adhere to a fixed sheet when they meet it ; many of these are the giant, often multinuclear, cells produced by aberrations of division (Roberts and Trevan, 1960*b*). We have a film sequence that shows a stable sheet being formed as a result of several successive divisions in an initially small group of cells in contact. The daughter cells from each division adhered firmly and promptly to their neighbours in the sheet ; nearby were several larger very actively amoeboid tumour cells that adhered neither to each other nor to the growing sheet.

If the culture fluid is removed and replaced with fresh medium after the formation of fixed cell-sheets and before the death of the culture there is an immediate renewal of amoeboid activity and the adhesions between cells in the sheet break. The cells form a sliding sheet or migrate apart ; if division has ceased it begins again. As time goes on, however, adhesions again form between cells and unite them in stable sheets. This happens more rapidly than it did in the fresh culture, i.e., in about 10 as against 30 hours ; the culture is now more crowded than it was when fresh, and the more crowded a fresh culture is the more quickly stable sheets are formed. A stable sheet re-formed after feeding the culture usually breaks up again at the next feed, but less completely, and it re-forms even more quickly than before. If the medium is renewed every day, stable sheet formation may be postponed at least until the point when the culture becomes so crowded that many cells die in spite of the frequent changes of medium. Cells in contact remain in sliding sheets throughout.

On three occasions stable sheets have been subjected to the replacement of the culture fluid with medium from which the glucose has been omitted. The sheets remained stable and adhesions between the cells did not break.

### DISCUSSION

When the cells of this ascites tumour derived from a well differentiated murine epithelioma are maintained in culture on a glass surface in a good state of nutrition they flatten, adhere to the glass, and migrate upon it. When they meet they slide around but not over one another, remaining in a monolayer, but they do not cohere. When pseudopodia of two cells meet, one of the pseudopodia may inhibit the activity of the other, as though it were " dominant ". Such

"dominance" is however often repeatedly reversed. This behaviour is not contact inhibition, described by Abercrombie and Heaysman (1954) as the normal behaviour of fibroblasts in culture, and as responsible for the maintenance of a monolayer: contact inhibition is defined as being associated with cell cohesion, and is believed to depend upon it. Abercrombie, Heaysman and Karthauser (1957) later reported that sarcoma cells failed either to show contact inhibition or to remain in a monolayer when confronted with other sarcoma cells or with fibroblasts. Thus the behaviour of Epithelioma 255 cells when they form non-coherent monolayers may be regarded as intermediate between these two extremes; they inhibit one another's movements more than sarcoma cells do, but do not cohere. This is reminiscent of the observations of Weiss and Taylor, quoted by Weiss (1958) of aggregates of normal epithelial cells. Moscona (1957) had found that suspensions of dissociated embryonic epithelium taken at a stage when differentiation was established would reaggregate according to histological type, even if the cells were from different species. Weiss and Taylor made cinematographic records of the re-aggregation process in order to discover what part if any was played by specific adhesion between cells of like type. Weiss concluded that "Cells in homologous, as well as heterologous, groups keep shifting about one another in a manner which rules out stable mutual attachments as a primary event in aggregation. The only difference between the homologous and heterologous groups seems to be that homologous cells tend to retain mutual contact along a broad front whereas heterologous combinations tend to break up soon after contact". He illustrates the break-up of a heterologous combination by a photograph of a pseudopodium of a liver cell within a smooth concavity on the surface of a lung cell; this strongly resembles what may happen when two cells of Epithelioma 255 meet; on the other hand, these epithelioma cells also form aggregates, which we have called "sliding sheets", which resemble the aggregates of homologous epithelium seen by Weiss and Taylor, in that "they retain contact along a broad front" without cohering.

The "dominance" of one pseudopodium over another might be explained in the following way. A cell puts out a pseudopodium by virtue of its contact with, and adherence to, the substrate. The advancing edges of opposed pseudopodia are thin and sharp; when they meet either edge may undercut the other and loosen it from the substrate so that it retracts elastically and progressively from the edge inwards. Further advance of the undercutting pseudopodium detaches more of the other from the substrate; if this advance ceases the undercut cell margin has an opportunity to spread out again and may undercut the other cell in turn. It may be a matter of chance which pseudopodium first undercuts the other. Once a cell border is undercut it becomes blunter and is thus less likely to undercut the other cell border until it can spread out again; this it cannot do until there is a clear space on the substrate to which it can adhere. The "dominance" we have seen applies at present only to pseudopodia on a glass substrate; we have so far not observed the behaviour of these cells in contact on other substrates or floating free.

If the medium in a culture of Epithelioma 255 ascites cells at the "sliding sheet" stage is not replenished, the cells begin to form more and more lasting adhesions to one another without at first any decline in migratory activity, so that they may move over the substrate in small compact groups. This shows that the formation of sheets of coherent cells is not entirely attributable to the gradual lessening of migratory activity that in the end accompanies relative starvation. This behaviour is analagous to contact inhibition. Abercrombie, Curtis and Karthauser (1958) have reported that some failure of contact inhibition may be produced in cultures of fibroblasts by a high concentration of embryo extract. Our observations of Epithelioma 255 also suggest a connection between nutrition and contact behaviour. There is no embryo extract in our medium, but the administration of medium containing little or no glucose to a stable sheet of cells of Epithelioma 255 does not cause it to break up ; this may indicate that the formation of intercellular adhesions is due to a reduction, by consumption, of the glucose available. Alternatively the cohesiveness may be induced by the consumption of some other nutrient or the production of some metabolite, and possibly the cells fail to part company when fed with medium lacking glucose because they have not enough energy to migrate apart ; the effect of glucose-free medium on cells in the early, actively migrating cohesive state has not yet been tried.

Dabrowska-Piaskowska (1959) applying Moscona's techniques to tumours found that cells of a murine mammary tumour reduced to a single-cell suspension with trypsin would re-aggregate into a structure resembling that of the tumour *in vivo*, when cultivated on glass or on a plasma clot. The sheets formed by Epithelioma 255 cells might be considered as analogous attempts at reconstruction, indeed, they show intercellular bridges which give them an at least superficial resemblance to prickle cells. The dependence upon nutrition *in vitro* of this apparent differentiation of the ascites form of Epithelioma 255 is of interest in that further study of the factors involved may throw some light on the mechanisms whereby the cells of a malignant tumour *in vivo* may vary in invasiveness or remain dormant for long periods.

### SUMMARY

Cells of a murine ascites epithelioma when cultured on a glass surface in a medium consisting of calf serum, lactalbumin hydrolysate, salts, bicarbonate, and glucose adhere to the glass and move about on it. When two cells meet one may inhibit the pseudopodial activity of the other, but they do not cohere at this stage ; at a later stage, if the culture medium is not renewed, adhesions are formed between cells which eventually unite them into stable sheets resembling normal epithelium. Cells in these sheets may be connected by intercellular bridges like those between prickle cells. The sheets grow by accretion and by division of the cells in them. This behaviour is likened to the reconstruction of normal tissues and of tumours from dissociated cells in culture that has been observed by other workers.

The formation of stable sheets may be prevented or reversed by renewing the culture medium, and there is a little evidence that the medium must contain glucose in order to do this. The dependence of apparent differentiation upon environmental factors, possibly nutritional, justifies further study in an attempt to elucidate some of the mechanisms responsible for the variation in invasiveness of tumours *in vivo*.

We are very grateful to Dr. Honor Fell, F.R.S. for her interest in this work, and for her help with its presentation.

### REFERENCES

ABERCROMBIE, M., CURTIS, A. S. AND KARTHAUSER, H. M.—(1958) *Rep. Brit. Emp. Cancer Campgn*, **36**, 507.
*Idem* AND HEAYSMAN, JOAN E. M.—(1954) *Exp. Cell. Res.*, **6**, 293.
*Iidem* AND KARTHAUSER, H. M.—(1957) *Ibid.*, **13**, 276.
DABROWSKA-PIASKOWSKA, KRYSTYNA.—(1959) *Ibid.*, **16**, 315.
MOSCONA, A.—(1957) *Proc. nat. Acad. Sci., Wash.* **43**, 184.
ROBERTS, D. C. AND TREVAN, D. J.—(1960*a*) *J. R. micr. Soc.*, **79**, 361.—(1960*b*) *Brit. J. Cancer*, **14**, 716.
WEISS, P.—(1958) " International Review of Cytology, vii, 391 ; Ed. Bourne and Danielli, New York (Academic Press).